# Evaluation of the Impact of Genetically Modified Cotton After 20 Years of Cultivation in Mexico

**DOI:** 10.3389/fbioe.2018.00082

**Published:** 2018-06-22

**Authors:** Martha G. Rocha-Munive, Mario Soberón, Saúl Castañeda, Esteban Niaves, Enrique Scheinvar, Luis E. Eguiarte, David Mota-Sánchez, Enrique Rosales-Robles, Urbano Nava-Camberos, José L. Martínez-Carrillo, Carlos A. Blanco, Alejandra Bravo, Valeria Souza

**Affiliations:** ^1^Departamento de Ecología Evolutiva, Instituto de Ecología, Universidad Nacional Autónoma de México, Ciudad de México, Mexico; ^2^Departamento de Microbiología Molecular, Instituto de Biotecnología, Universidad Nacional Autónoma de México, Cuernavaca, Mexico; ^3^Department of Entomology, Michigan State University, East Lansing, MI, United States; ^4^Weed Management Independent Advisor, Río Bravo, Mexico; ^5^Facultad de Agricultura y Zootecnia/Facultad de Ciencias Biológicas, Universidad Juárez del Estado de Durango, Gómez Palacio, Mexico; ^6^Dirección de Recursos Naturales, Instituto Tecnológico de Sonora, Ciudad Obregón, Mexico; ^7^Biology Department, University of New Mexico, Albuquerque, NM, United States

**Keywords:** Bt cotton, center of origin, environmental impact, GMO, herbicide, Mexico

## Abstract

For more than 20 years cotton has been the most widely sown genetically modified (GM) crop in Mexico. Its cultivation has fulfilled all requirements and has gone through the different regulatory stages. During the last 20 years, both research-institutions and biotech-companies have generated scientific and technical information regarding GM cotton cultivation in Mexico. In this work, we collected data in order to analyze the environmental and agronomic effects of the use of GM cotton in Mexico. In 1996, the introduction of Bt cotton made it possible to reactivate this crop, which in previous years was greatly reduced due to pest problems, production costs and environmental concerns. Bt cotton is a widely accepted tool for cotton producers and has proven to be efficient for the control of lepidopteran pests. The economic benefits of its use are variable, and depend on factors such as the international cotton-prices and other costs associated with its inputs. So far, the management strategies used to prevent development of insect resistance to GM cotton has been successful, and there are no reports of insect resistance development to Bt cotton in Mexico. In addition, no effects have been observed on non-target organisms. For herbicide tolerant cotton, the prevention of herbicide resistance has also been successful since unlike other countries, the onset of resistance weeds is still slow, apparently due to cultural practices and rotation of different herbicides. Environmental benefits have been achieved with a reduction in chemical insecticide applications and the subsequent decrease in primary pest populations, so that the inclusion of other technologies—e.g., use of non-Bt cotton- can be explored. Nevertheless, control measures need to be implemented during transport of the bolls and fiber to prevent dispersal of volunteer plants and subsequent gene flow to wild relatives distributed outside the GM cotton growing areas. It is still necessary to implement national research programs, so that biotechnology and plant breeding advances can be used in the development of cotton varieties adapted to the Mexican particular environmental conditions and to control insect pests of regional importance.

## Introduction

Cotton is one of the most important natural sources for fiber, oil, and seeds for livestock feeding. All the cotton produced in the world is obtained from four domesticated species of the *Gossypium* genus of the Malvaceae family. With 18 species, Central, and South America are the richest regions in *Gossypium* species globally, being Mexico one of the most diverse countries with 14 different species. The northeast of Africa and the southwest of Arabia also have 14 different species and Australia has 17 species (Cronquist, 1981; Fryxell, 1992; Percival et al., 1999).

An outstanding feature of cotton domestication is that it occurred simultaneously in different continents from local cotton wild ancestors. This process of parallel and convergent domestication occurred for the species *Gossypium hirsutum* in Mexico, *G. barbadense* in Peru, *G. arboreum* in Sudan and *G. herbaceum* in Pakistan. In each of these four cases, the unique properties of cotton fiber useful to make ropes and textiles were noticed thousands of years ago. From these four species, *G. hirsutum*, commonly referred to as Mexican cotton or highland cotton, is the most widely planted, accounting for 90% of world production. This is relevant, since Mexico is an important center of origin and domestication of many other cultivated crops, such as corn, squash, pumpkin, bean, and chilies. Currently in Mexico several native cotton species are present, including the wild relatives of *G. hirsutum*. The highest concentrations of wild cotton relatives are located in the southeast region of the country, the only place where *G. hirsutum* is found as a common species in the native flora (Coppens d'Eeckenbrugge and Lacape, 2014).

Before the deployment of GM technology, cotton production was associated to high environmental, economic, and sanitary costs due to the necessity of large amounts of pesticide applications. A different strategy was necessary to improve yields, thus technology involving GM cotton cultivars with inserted genes that confers resistance to lepidopteran pests and to herbicides was adopted by the growers (Deguine et al., 2008; Benbrook, 2012).

In Mexico as in other parts of the world, the cultivation of cotton was characterized by the application of large quantities of chemical insecticides. For example, in the 1970s decade, cotton cultivation required almost 20 applications of chemical insecticides from the plant emergence to harvest, since cotton plants must be protected from insect attack when the plant emerges, until the profitable bolls open (a period that lasts ~20 weeks). In the middle of the Twentieth century, at the peak of cotton production in Mexico, the cotton area that was planted reached 900,000 hectares with 2 million bales produced per year (the term “white gold” was used at that time to describe cotton). Years later, the increasing pest pressure and high doses of pesticides resulted in the evolution of insect resistance to chemical insecticides. In addition, reductions in international prices of the fiber resulted in a production decline due to unsustainable operating costs (Martínez-Carrillo and Díaz-López, 2005; Martínez-Carrillo, 2015).

In 1996, GM cotton was for the first time commercially planted in Mexico as well as in five other countries (James, 2016), due to the impossibility of cultivating conventional cotton in areas of severe pest pressure (Terán-Vargas et al., 2005). Since then, a total of 15 countries have commercialized GM cotton (Argentina, Australia, Burma, Brazil, Burkina Faso, China, Colombia, Costa Rica, United States, India, Mexico, Paraguay, Pakistan, South Africa, and Sudan). In Mexico, the increase in GM cotton adoption was gradual (Martínez-Carrillo, 2005), and since 2008 the 96% of the area cultivated with cotton was GM cotton (Purcell et al., 2008).

Nevertheless, the area planted with GM cotton in Mexico has fluctuated, depending on international fiber prices, input costs and the prevalence pests, weeds, and diseases. The main cotton production areas of Mexico are located in the northern region of the country. This region has an arid climate and growers used irrigation systems. These areas of cotton production are not in close proximity to areas containing wild relatives of cotton, as stated in the Mexican law (CIBIOGEM, 2018).

The transformation events or transgenes that have been authorized in Mexico since 1996 confer two main traits, one is the tolerance to herbicides and the other is the resistance to lepidopteran pests. In the first case, plants are tolerant to herbicides such as glyphosate (Nida et al., 1996), ammonium glufosinate (Blair-Kerth et al., 2001) and dicamba (Cahoon et al., 2015) that are used to combat weeds. In the second, resistance to lepidopteran pests is due to the insertion of *cry* genes from the bacterium *Bacillus thuringiensis* (Bt) that confers resistance to larval stages of different lepidopteran pest such as *Pectinophora gossypiella, Helicoverpa zea, Heliothis virescens* (Benedict et al., 1993), and *Spodoptera exigua* (Wilson et al., 1992; James, 2016).

**Figure 1:**
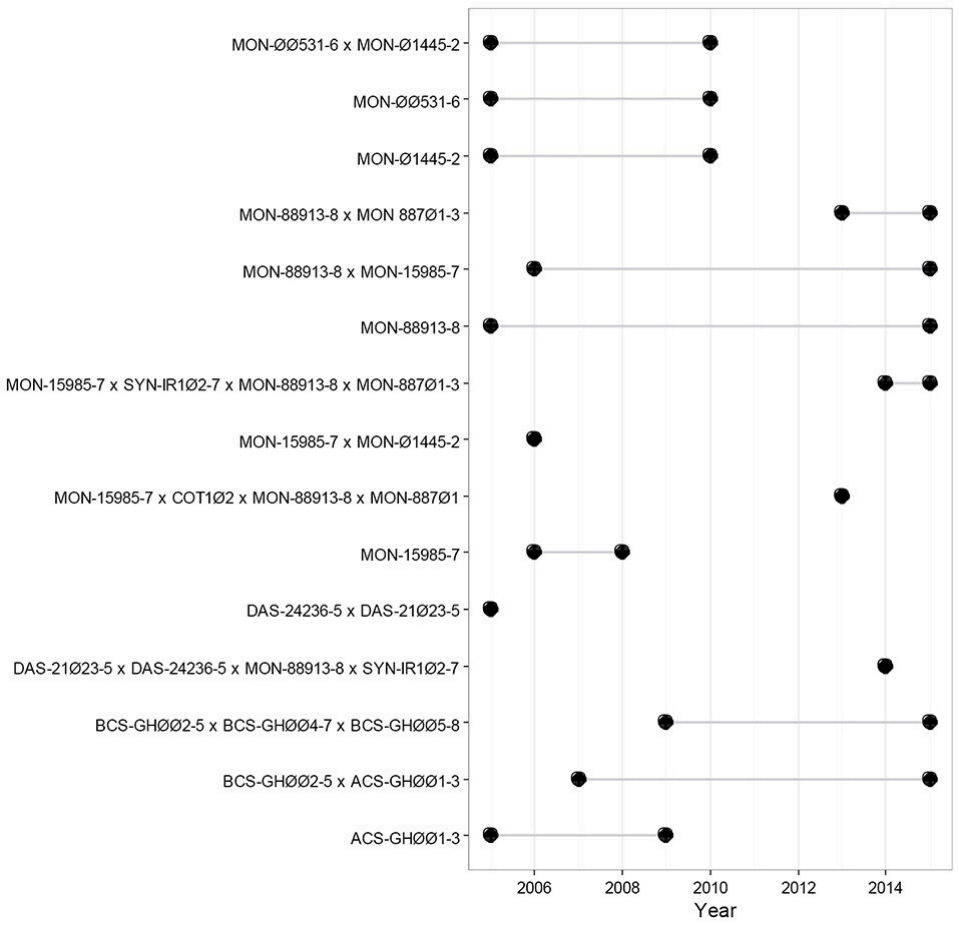
Timeline for GM cotton transformation events released in Mexico. Black dots indicate the first and last year that each release was requested. The figure shows the different GM cotton released applications that have received a permit in Mexico, from 2005 until 2015 (CIBIOGEM, 2018).

In Mexico, the “Biosafety Law of Genetically Modified Organisms” regulates the cultivation of GM cotton and other biotech crops in a step-by-step and case-by-case basis. The different steps refer to the different stages of release: experimental, pilot and commercial plantings. Prior to the commercial release, the authorities evaluate the results of the experimental and pilot (semi-commercial) scale releases, carrying out risk assessment studies and examining the experimental results, as well as the compliance and effectiveness of the biosafety measures (DOF, 2005). Academic institutions must endorse the research carried out in Mexico. A total of 15 GM cotton release events were requested from 2005 to 2015, in 342 dossiers [Figure 1; (CIBIOGEM, 2018)].

The environmental risk assessment studies aim to identify potential damage to the environment where the level of risk is estimated, the potential negative effects are identified, and actions needed to reduce environmental risks are determined (EPA, 1998). In the case of the environmental risks associated with the release of agricultural GMOs, it is important to compare them with the risks associated to the agricultural practices used on conventional crops. This is why a “case by case” analysis should be performed, that is, to consider the modified organism, the intended use, and the likely environment and environmental conditions in which it will be grown. The risk assessment studies for the release of GM cotton in the case of Mexico included an evaluation of the risks of gene flow to wild relatives, the possible effect on non-target organisms, the risks of selection of resistant weeds to herbicides and the evolution of resistance to Cry proteins by the insect pests (SEMARNAT, 2018).

In this work, we present an updated analysis of the data available since the release of GM cotton in 1996. Two main hypothesis were questioned: the first hypothesis is if there is potential risk in gene flow to native species, while the second is if the use of GM-cotton in Mexico would result in a reduction of pesticides use and in higher yields.

## Methods

### Analysis of wild cotton species distribution

For the analysis of the wild cotton species distribution, we used the CONABIO database where 2,238 records were cured and verified (including 16 cotton species: *G. thurberi, G. armourianum, G. harknessii, G. davidsonii, G. aridum, G. raimondii, G. gossypioides, G. lobatum, G. laxum, G. trilobum, G. turneri, G. schwendimanii, G. lanceolatum, G. hirsutum*, and *G. barbadense*; CONABIO, 2018).

In order to assess the likelihood of gene flow, the cotton growing regions were characterized and a distribution model of wild *G. hirsutum* was constructed. The environmental characteristics of these cotton growing regions were identified by a classification tree, using as covariates of 19 current bioclimatic layers (Hijmans et al., 2005), 12 solar radiation layers (WorldClim), terrain slopes and ruggedness index.

### Development of an ecological niche models

To elaborate ecological niche models (ENM) of two different scenarios of cultivated cotton (without volunteers and with volunteers), we used a database constructed with 259 unique presence records of GM cotton plots and 17 records of cotton volunteers reported by several volunteer monitoring campaigns carried out in the cotton growing regions. Records from plots in the Northeast region (Tamaulipas) were not available and were not included in the analysis.

Nineteen current bioclimatic layers were downloaded from the WorldClim 1.4 data set (Hijmans et al., 2005) and six topographical layers from the HYDRO1k Elevation Derivative Database (available at: http://lta.cr.usgs.gov/HYDRO1K), using a resolution of 30 arc-s (ca. 1 km).

Maxent 3.3.3e (Phillips et al., 2006) runs were performed, one for each scenario. Each run included 30 replicates using the logistic model, and 20% random test by bootstrap. All the distribution models were evaluated using AUC scores (0.98 with and without volunteers). The models were transformed into binomial data, with a total presence value as the cut-off for each scenario (0.01 without volunteers and 0.15 with volunteers).

### Surveys of cotton farmers

In Mexico, cotton farming is commonly managed by the owner of the land or the farmer that uses it, and a “technical advisor,” that is a professional pest control crop advisor.

In order to determine the perception of the Mexican farmers on the impacts of planting of GM cotton, a survey was designed and applied to 167 farmers in 20 municipalities of the main cotton-producing states. The objectives of the survey were to identify factors associated with the use of GM cotton in Mexico, to know the willingness of farmers to use this biotechnology and the perception of benefits or problems that they have observed, to identify changes in yields, production costs, control of pests, handling, and use of pesticides from the transition from conventional to GM cotton and to evaluate the indirect effects of the use of this technology on the environment and in human health. The survey was designed according to the methodology of agricultural surveys with multiple sampling frames and the sample design for the study of rural organizations in Mexico (Kish, 1990; González-Villalobos and Wallace, 1998). The margin of error of this survey was ±7.46% with a total estimated population of 5,000 cotton farmers and a confidence level of 95% (Survey System, 2018).

### Surveys of technical advisors

A survey was applied to 165 technical advisors specialized in cotton management. This survey was based on Shaw et al. (2009), to assess the impact of GM-crops with tolerance to glyphosate. Questions related to the pest management were also added.

**Figure 2:**
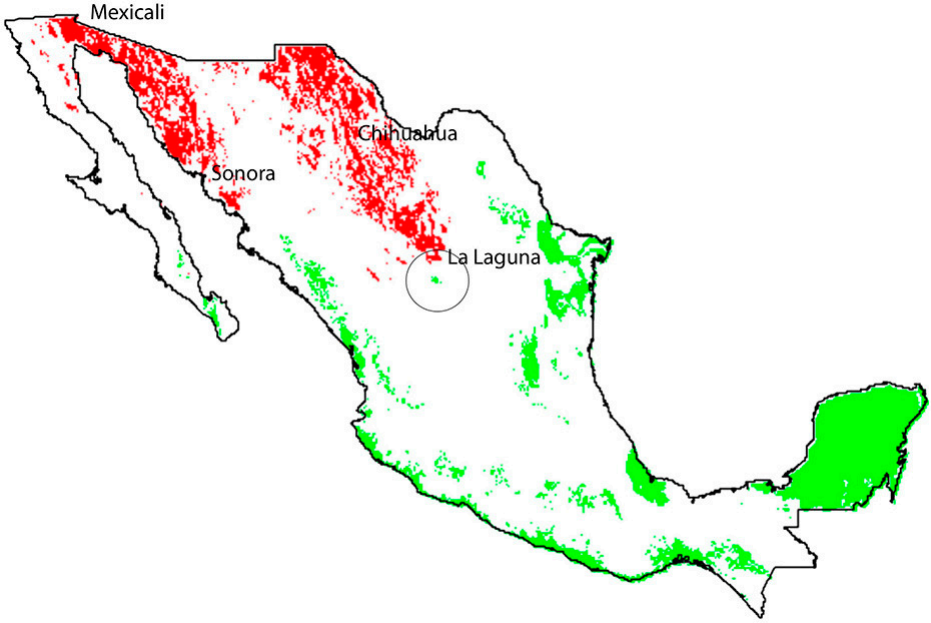
Climatic suitability model for geographical space projection regions for wild cotton *G. hirsutum* (green dots) and cultivated GM cotton regions (red dots). Regions do not overlap, but show proximity in the area known as “La Laguna” (black circle).

The technician advisors' sample size was: Mexicali (*n* = 46); Chihuahua (*n* = 39); and La Laguna (*n* = 80) (Figure 2). The margin of error of this survey was ± 7.5% and a confidence level of 95% (Survey System, 2018).

## Results and discussion

### Exploring the first hypothesis: gene flow from cultivated cotton (conventional or transgenic) to wild relatives

Since Mexico is a center of the origin and diversification of *G. hirsutum*, one of the main environmental concerns for the release of GM cotton was the possibility of transgene flow to native cotton populations (Ellstrand, 2002, 2012; Ellstrand et al., 2013).

In Mexico there is a continuum of *G. hirsutum* cotton varieties that range from wild, feral and locally domesticated to improved varieties, therefore the potential for gene flow among them exists if they coexist in the same area. To assess such risk, it is necessary to know the geographic distribution patterns of the different varieties, and also the dispersal mechanisms of the species. The geographical distribution of wild populations and cultivated cotton was taken into account in the risk assessment evaluation and the geographical separation constitutes one of the conditions in México for the release of GM cotton into the environment and before sowing field visits were done to identify the possible presence of wild cotton relatives (BCH, 2018; SAGARPA, 2018).

The geographical overlap between native species distribution and the region in which GM cotton is currently planted is minimal, according to the records of the “National Commission for Knowledge and Use of Biodiversity” (CONABIO, 2018). The delimited GM cotton growing regions correspond to semi-arid regions (Figure 2, red dots) that do not geographically overlap with the area of climatic suitable zones of wild *G. hirsutum*. However, they are close to the La Laguna region (Figure 2). Few GM cotton regions were not included in the analysis either due to security issues or restrictions in technical support (i.e., North of Tamaulipas, Valleys of Yaqui, and Mayo, and Planicie Huasteca), all of them coincided with the climatic suitability zones of *G. hirsutum*. Nevertheless, according to the National Statistics (INEGI, 2012), Tamaulipas is the state with less cotton production in the country and the Yaqui valley as well as the Planicie Huasteca are not even in the statistics of cotton production.

For gene flow through pollen to occur, it is not only required that the plants coexist in the same area and that they are compatible, but also that the pollen containing transgenes is dispersed via pollinators. In the case of cotton, the rate of cross-pollination (the probability that a plant is pollinated with pollen from other plant) is 10% or less, since 90% of the plants resulted from self-pollination (Meredith and Bridge, 1973; Llewellyn and Fitt, 1996; Sen et al., 2004; Van Deynze et al., 2005; Zhang et al., 2005). It was also reported that, in cases where cross-pollination by bees occurs, it significantly decreases with the distance between plants. High cross-pollination probability occurs only when plants are located in close proximity (Umbeck et al., 1991; Yan et al., 2015). Moreover, the cross-pollination rate depends, to a large extent, on the climatic and ecological condition that determine, for example, the patterns of activity and abundance of insect species carrying out pollination and pollen flow (Llewellyn et al., 2007).

However, in our study we observed that the most imminent risk of gene flow is not by pollen, but by seeds spilled during transportation. Cotton-seeds can be efficiently dispersed by either wind or water. During several field visits to the cotton productions areas, it was observed that there is a very strict control and biosafety measures during the movement of the GM cotton-seeds from the seed-companies to the fields. The GM seeds arrive in closed packages and closed vehicles. However, after the harvest, such controls relaxed, and the seeds are transported to the gins in open vehicles that spill seeds in the roads. Volunteer plants can grow from spilled seeds and have been observed in the edge of roads. Sanity authorities and seed companies are in charge of removing the volunteer plants, but unnoticeable escapes are always possible.

**Figure 3:**
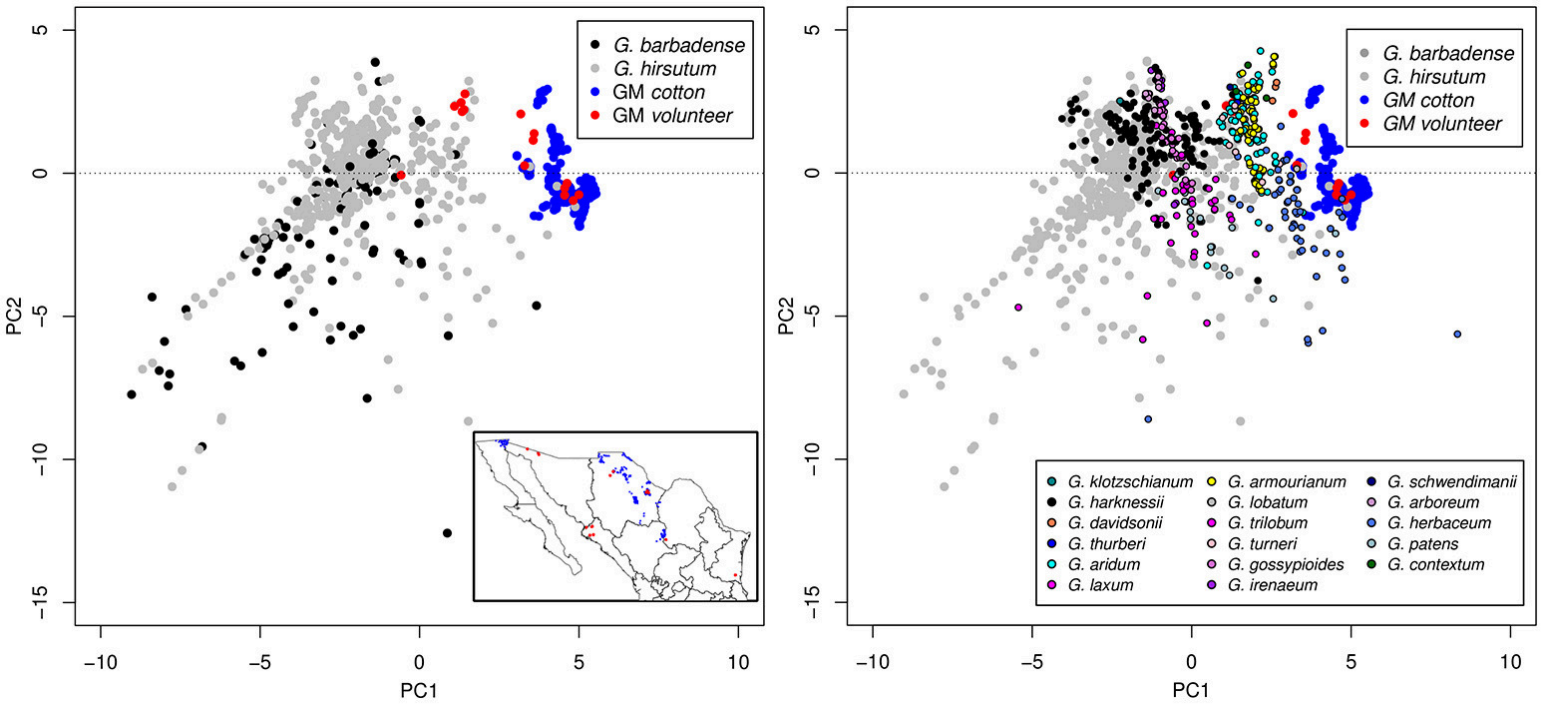
Principal Component Analysis (PCA) of environmental conditions of the analyzed cotton records (wild, GM, and volunteer). Conditions in which GM cotton is planted (blue dots) are very restrictive and differentiable from the rest of the cotton species (wild in black, gray, and colors). GM volunteers are represented with red dots.

**Figure 4:**
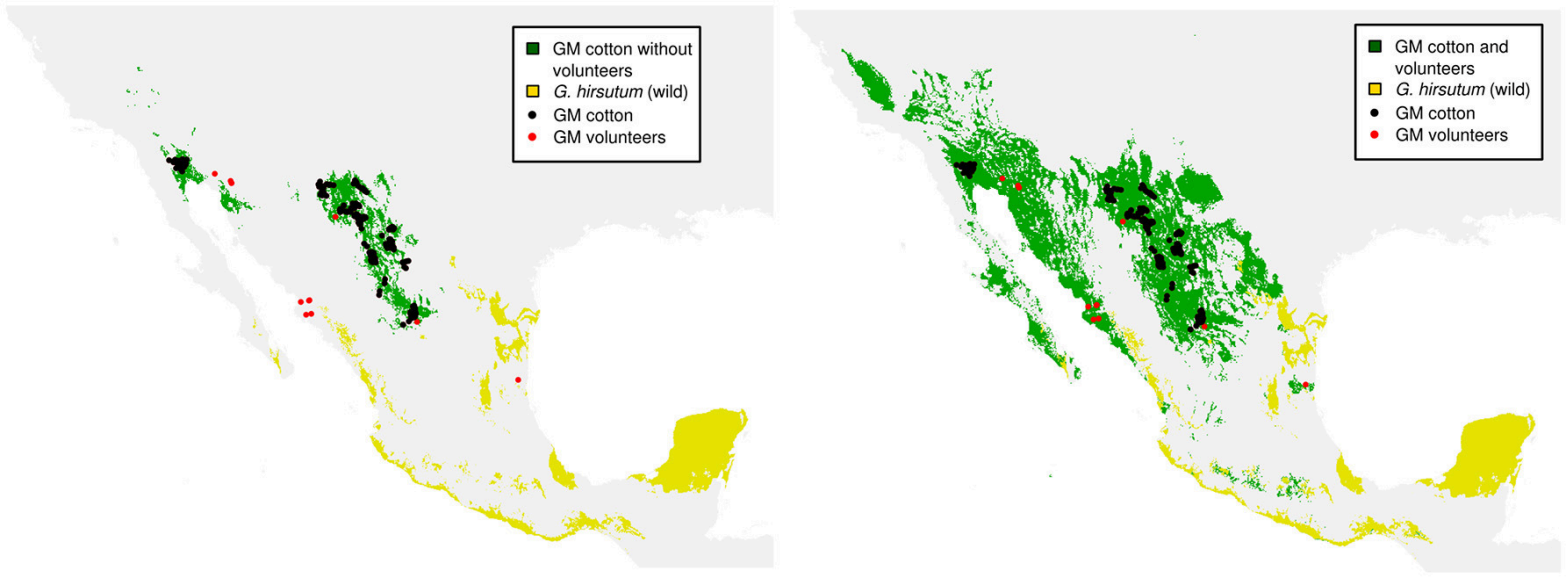
GM cotton distribution models adding the presence of volunteers. In green: potential distribution of GM cotton. In yellow: potential distribution of wild cotton. Black dots indicate the records of GM cotton plots while red dots indicate the records of volunteer plants used for the elaboration of the models.

From the two scenarios of cultivated cotton (without volunteers and with volunteers), we further elaborate an ENM as described in Methods. Figure 3 shows the Principal Component Analysis (PCA) of environmental conditions of the analyzed cotton records (wild, GM, and volunteer). It can be seen that the conditions in which GM cotton is planted (blue dots) are very restrictive and conditions are clearly differentiable from the rest of the cotton species (wild in black, gray, and colors). However, the presence of GM volunteers (red dots) in environments other than GM growing regions demonstrates the environmental plasticity of GM cotton, and broadens the environmental component of the GM cotton niche toward the environmental space occupied by wild species. In Figure 4 we show the potential distribution of GM and wild cotton. According to the models describing the two possible scenarios (without and with volunteers), this figure shows that the presence of volunteers significantly expands the niche of GM cotton in its geographic component (Figure 4).

It is important to mention that Wegier et al. (2011) reported the existence of gene flow at long distances between cultivated and wild populations of *G. hirsutum*, by the identification of recombinant proteins in wild populations of cotton. These authors proposed that the gene flow may be possible through the dispersion of seeds (Wegier et al., 2011). Hence, it is necessary to follow up the monitoring of hybrid populations and implement sensitive methods such as RT-PCR and digital-PCR to evaluate in detail the changes in transgene frequencies in these populations (Holst-Jensen, 2009; Fraiture et al., 2015; Randhawa et al., 2016).

### What do people that work with the GM cotton in Mexico think

#### Surveys of cotton farmers

Overall, farmers pointed out that the use of GM cotton resulted in better pest control and easier pest management. Also, higher yields of GM cotton were generally mentioned. The reasons for stopping the planting of non-Bt conventional seed include difficulty for controlling pests and high costs of insecticides. According to the opinion of the farmers, GM cotton showed higher yields and required less use of insecticides and crop management. Nevertheless, according to farmers' opinions GM cotton-seeds are expensive and the use of herbicides is higher. In addition, farmers agreed that the highest yields of GM cotton are due to better seed quality and favorable weather conditions.

Cotton is planted in the arid areas of northern Mexico, where adverse weather conditions are prevalent, including the lack of water, extreme temperatures, drought, and frost. Inputs such as special planting equipment, irrigation, and fertilizers result in high production costs. In addition, an increase in seed prices, machinery, and fuels in recent years exacerbated the production costs.

The high operation costs as well as fluctuations in international fiber prices, led to a large fluctuation in the total cotton area planted. For instance, in 2016 the total cotton area in Mexico was reduced to 104,000 ha, due to the decrease in international prices and the increase in input costs. However, the cotton area was doubled to 210,000 ha in 2017 due to an increase in international fiber prices. The decrease in grain prices could be another important factor that favors cotton growing for some farmers.

Despite the cost of production, 80% of the farmers are highly satisfied with the use of the GM varieties, since the lepidopteran pests are controlled and excellent weed control is obtained. The remaining 11% of farmers are moderately satisfied, and 9% are not satisfied. Ten percent of the farmers considered that GM cotton is not profitable.

Interestingly, 40% of the farmers would be willing to plant conventional seeds if available in Mexico (conventional seeds are not produced now in Mexico), because it is assumed by these farmers that those seeds would cost less. Furthermore, due to current pest populations observed for the past few years, they considered that current pests are not necessarily controlled by GM varieties.

From the point of view of the effects on human health, farmers have a positive perception about the adoption of GM cotton. They believe that the intoxication cases due to chemical pesticide exposure have been reduced with the adoption of GM cotton. They reported less intoxication cases due to a lower use of chemical insecticides (Nava-Camberos et al., unpublished results).

#### Surveys of the technical advisors

In order to analyze changes in pest and weed management after the adoption of GM cotton a survey was applied to 165 technical advisors specialized in cotton management.

With respect to the management of weeds and herbicides, the responses of the technicians indicated that glyphosate is practically applied to the entire cotton growing area in Mexico at least once during the production cycle. The main weed species associated with cotton are field bindweed *Convolvulus arvensis L*., annual morning glories *Ipomoea hederacea* Jacq. and *Ipomoea purpurea* (L.) Roth, palmer amaranth *Amaranthus palmeri S*. Wats, johnsongrass *Sorghum halepense* (L.) Pers. and various annual grasses, mainly barnyardgrass *Echinochloa colona* (L.) Link.

According to these surveys, weed management in cotton in Mexico generally consists of the application of glyphosate that is complemented by deep tillage for soil preparation and in-row cultivation in more than 90% of the cotton area. The application of other herbicides in addition to glyphosate in pre-planting and pre-emergence is done in about 21% of the area where trifluralin represents the most used herbicide in these early applications.

Technicians indicated that problems associated with weed management were reduced in Mexicali and La Laguna, but they were increased in the state of Chihuahua, where the control of weeds with glyphosate was qualified as low. Sixty two percent of the technicians indicated that they have observed changes in the response of weeds to glyphosate. This response of the weeds implies the need of a dose increase of herbicides in order to have an effective control in the most difficult weeds. Nevertheless, 85% of the technicians are currently carrying out management practices to prevent the selection of glyphosate-resistant weeds, focusing mainly to in-row cultivation, hand weeding and crop rotation.

Before the use of Bt cotton, the Lepidoptera complex (*P. gossypiella, H. zea, H. virescens*, and *S. exigua*) comprised the majority (ca. 60%) of the total reported pests; followed by sucking insects (whitefly, *Chlorochroa ligata* and *Lygus*; ca. 20%). The reported insects list is presented in Table 1, where it is observed this drastic drop in lepidopteran counts, while other insects such as aphids, mites, weevils, thrips, and whiteflies increased in counts by the technicians. The technicians consider that the pressure of the Lepidoptera complex was very high before the use of GM-cotton and now it has effectively been reduced.

**Table 1:** Insects reported by technicians as important pests in all regions.

**Insects reported by technicians**	**Before**	**After**	**Effect**
**DECREASE IN COUNTS**			
Cotton bollworm (*Helicoverpa zea*)	116	6	−110
Pink bollworm (*Pectinophora gossypiella*)	103	6	−97
Beet armyworm (*Spodoptera exigua*)	17	7	−10
Cotton leaf perforator (*Bucculatrix thurberiella*)	6	0	−6
Tobacco budworm (*Heliothis virescens*)	3	0	−3
Fall armyworm (*Spodoptera frugiperda*)	2	1	−1
Cabbage looper (*Trichoplusia ni*)	2	1	−1
Cotton fleahopper (*Pseudatomoscelis seriatus*)	1	0	−1
**INCREASE IN COUNTS**			
Yellow sugarcane aphid (*Melanaphis sacchari*)	0	1	1
Stink bug (*Nezara viridula*)	1	2	1
Red spider mite (*Tetranychus* sp.)	0	3	3
Cotton aphid (*Aphis gossypii*)	3	13	10
Boll weevil (*Anthonomus grandis*)	46	57	11
Conchuela bug (*Chlorochroa ligata)*	23	47	24
Other Hemipterous plant bugs	23	50	27
Thrips (*Frankliniella occidentalis* y *Thrips tabaci*)	4	33	29
Lygus bug (*Lygus lineolaris*)	5	38	33
Whitefly (*Bemisia tabaci*)	21	71	50

*Numbers indicated the number of times that a technician mentioned the name of the insect as important before the deployment of Bt cotton and after it. The effect was measured by the subtraction of both values. Negative values indicate a decrease in the number of times reported and positive values indicate an increase in the times reported*.

After 20 years of using Bt-cotton, the interviewed technical advisors have observed drastic changes in the composition of insect pest species. Currently, the most important are *Anthonomus grandis, C. ligata, Bemisia tabaci*, several species of sucking insect pests, and thrips. The Lepidoptera complex represented only up to 5% of the reported pests (mentioned by 0, 0, 0, and 5% of the technical advisors in Mexicali, Sonora, La Laguna, and Chihuahua, respectively) while the sucking insect pests comprised around 73% (60, 60, 80, and 95% of the survey in Sonora, La Laguna, Chihuahua, and Mexicali, respectively). Due to environmental differences in the cotton growing regions of Mexico, it is difficult to rank the overall importance of pests. For example, whiteflies are of primary importance in Mexicali, Sonora, and La Laguna, but in Chihuahua, they are considered a secondary pest. Conchuela (*C. ligata*) is still considered the primary pest in La Laguna and Chihuahua, but it is not a concern in Mexicali and Sonora. *A. grandis* once a menacing pest throughout Mexico, currently is only important in La Laguna and Sonora, but in Mexicali and Chihuahua this pest is eradicated. This eradication is due to the joint *A. grandis* eradication Mexico-USA program. After using Bt-cotton, *P. gossypiella, H. virescens*, and *Bucculatrix thurberiella* now have very low population levels in the different cotton regions. *H. zea* and *S. exigua* are currently considered pests of secondary importance in all cotton areas (Table 1).

Regarding the number of total insecticide applications, the technicians reported a significant decrease due to the use of GM cotton. Due to the effectiveness of Bt-cotton, and its high rate of adoption in most of the growing areas, in Chihuahua and La Laguna the synthetic insecticides sprays have been reduced to 3.5 and 5.0 applications, respectively, from the previous ~12 applications used in a crop season. Nevertheless, in other regions such as Mexicali and Sonora that showed high pressure of pests that are not targeted by Bt-cotton (whiteflies, *Lygus* bugs, and boll weevils) the insecticide sprays are still high.

### Exploring the second the hypothesis: effects and impacts of GM cotton cultivation in Mexico

Different lines of evidence indicated that the use of GM cotton has contributed to reducing the number of insecticide applications necessary to achieve adequate control of lepidopteran pests in the cotton regions of Mexico. Cotton is one of the crops in which the greatest amount of pesticides is applied in the world, so the alternative of using Bt-cotton represents an advantage from the environmental point of view (Abedullah et al., 2015). It is known that the use of pesticides can have negative impacts on the quality of water and soil, human health, aquatic species, and beneficial insects and other organisms (Boatman et al., 2004; Arias-Estevez et al., 2008; Athukorala et al., 2012).

According to most farmers, GM cotton in Mexico, despite its costs, is still economically profitable and is one of the main income sources in the municipalities where it is planted. In those places, GM cotton seems to ensure production, and prevent losses by lepidopteran insect pests, while reducing costs and labor activities as well as the use of vehicles to spray pesticides (Skevas et al., 2013). The impact on crop yield has also been significant since in Chihuahua, La Laguna and Mexicali the yield increments are 1.8, 2.4, and 3.7 bales per ha, respectively, which is equivalent to increases of $8,700, $11,500, and $17,700 Mexican pesos per ha.

It is difficult to illustrate the agronomic advances that the cotton industry has experienced in recent decades without also involving factors such as the improvement of seeds, the better use of water and fertilizers. Great effects are the result of better training of the agricultural technicians and government campaigns for crop health. Pest eradication is an additional benefit of this technology. For example, since 2007 it has not been necessary to apply insecticides against *P. gossypiella* in Chihuahua. It is calculated that the *P. gossypiella*-eradication program resulted in 1.7 million less liters of chemicals saving of more than 207 million Mexican pesos for cotton producers (CESAVECH, 2015).

Few studies have analyzed the effect on human health and the environment of GM cotton. Adoption of Bt-cotton reduced acute pesticide poisoning in farmers in China and India (Hossain et al., 2004; Kouser and Qaim, 2011). The compounds present in the pesticides used in conventional crops tend to accumulate in human tissues and are very dangerous for workers if the appropriate safety equipment is not used.

As mentioned before, different data and our surveys indicate that the intensity with which pesticides were used before GM cotton was very high. The intense use of broad-spectrum insecticides in conventional cotton was highly toxic, since those compounds affect many kinds of animals, including humans, and usually have high permanence in the field, affecting food chains of predators, parasitoids, and pollinator insects.

### Ecological and evolutionary aspects of GM cotton

#### Effect of Bt-cotton on non-target insects

Annual crops such as cotton require a field season comprised of 6–7 months and involve the intensive management of both weeds and insect pests. The Cry toxins produced by Bt that are expressed in different cotton events (Bt cotton) are specific to insects of the order Lepidoptera. These toxins are active against common cotton pests such as *P. gossypiella, H. zea, H. virescens*, and *S. exigua*. Thus, the control of other pests of different insect orders that attack cotton such as the coleopteran *A. grandis*, or the hemipteran *B. tabaci* or other insect pests still require applications of synthetic insecticides.

It is important to note that formulated insecticides based on Bt are used in integrated pest management (IPM) and organic agriculture because of their high specificity. Bt is also integrated into pest management, due to its biodegradable nature and ability to control specific pests, lacking impact on non-target organisms such as bees, parasitoid wasps, earthworms, beneficial true bugs, or predatory beetles, which do not possess an active target site (or receptor) where the Bt protein can interact (Pardo-López et al., 2013). The results of numerous studies with Bt toxins show that when non-target organisms are exposed to Bt toxins in similar amounts, or higher than those produced by the Bt-crops, they are not affected (Zwahlen et al., 2003; Ferry et al., 2005; Lu et al., 2010; Schuler et al., 2013). Among the most detailed studies are those in which a pest (e.g., an aphid, mite or worm) is fed on Bt-cotton and is consequently consumed or parasitized by a predator/parasitoid without any effect on the non-target insect (Zwahlen et al., 2003; Ferry et al., 2005; Lu et al., 2010; Schuler et al., 2013).

Due to the high effectiveness of Bt cotton against the most important lepidopteran pests, the damage induced by these Lepidoptera complex in Bt cotton is substantially smaller, or non-existent, when compared with the damage that they produced on conventional cotton if they were not controlled by chemical insecticides. However, the reduction of lepidopteran pests in Bt cotton may result in an increase of other cotton pests that are not controlled by Bt cotton. This phenomenon has been observed worldwide (Wang et al., 2006, 2009; Zhao et al., 2011) suggesting that secondary pests can occupy the resources previously used by lepidopteran insects. However, it was also reported that the lower use of chemical insecticides promotes the increase of natural enemies than can decrease populations of other non-target pests (Tian et al., 2015).

This increase of secondary pests apparently has been erroneously interpreted as an undesired effect of Bt cotton (Wang et al., 2008; Li et al., 2011; Zhao et al., 2011). Nevertheless, farmers generally control outbreaks of secondary pests with broad-spectrum insecticides. This practice, although effective against the target insects, also kills beneficial organisms.

It has also been shown that populations of non-target organisms may fluctuate in conventional cotton fields compared to those of Bt cotton, since the density of a pest may have consequences on the abundance of predators and parasitoids (Romeis et al., 2006). The reduced applications of the broad-spectrum pesticides may favor the increase of beneficial insect populations. However, a lower number of lepidopteran eggs and larvae in Bt cotton can affect the availability of food and hosts of natural enemies. Since the vast majority of these biological control agents have broad diets, the decrease in eggs, and larvae of lepidopteran insects affects their populations only temporarily (Theiling and Croft, 1988; Bradbury and Coats, 1989; Pisa et al., 2015).

Considering the ongoing controversy regarding the environmental impact of Bt cotton and particularly the scarce information on its effects on the diversity of the non-target insects under Mexican conditions, a study was carried out comparing arthropod populations in non-Bt and Bt cotton in the states of Durango and Coahuila (known as “La Laguna;” Nava-Camberos et al., unpublished results). Key target pests *H. zea* and *S, exigua* were only abundant in non Bt-cotton, while no differences were found in overall arthropod species composition and abundance between conventional and Bt-cotton areas. Among them, insects of three orders (Hemiptera, Thysanoptera, and Diptera) and three families (Aleyrodidae, Anthocoridae, and Thripidae) were the most abundant. At the trophic level, the total number of entomophagous and phytophagous insects was similar in both types of cotton. However, the non-Bt cotton presented a reduced diversity index, after several applications of insecticides (Nava-Camberos et al., unpublished results).

#### Evolution of resistance in insects

One of the most important economic risks of genetically modified crops is the evolution of resistance to Cry proteins by insects (Tabashnik et al., 2008) and to herbicides by weeds (Powles, 2008; Heap, 2018). In the case of Bt crops, the evolution of resistance to these crops has already been reported in different parts of the world in Bt corn and Bt cotton that express a single Cry protein (Tabashnik et al., 2008, 2013) or two Cry proteins (Jurat-Fuentes et al., 2003), although there has been no report of such resistance in Mexico (Tamez, 2010; Aguilar-Medel et al., 2017; Mota-Sanchez and Wise, 2018).

One strategy to delay the evolution of resistance is the deployment of “refuges,” which consist of plots with non-Bt plants near GM crops (Georghiou and Taylor, 1977; Gould, 1998). For the refuge strategy to be effective, insect resistance should be recessive (Carrière et al., 2010). This means that a resistant insect must carry two copies of the resistant allele. Heterozygous individuals with just one copy of the recessive allele are sensitive to a Cry toxin present in Bt cotton, and only homozygous individuals carrying two copies of the resistant alleles survive on the Bt plants. Therefore, the refuge has the purpose of maintaining a healthy population of susceptible insects. The idea is that when homozygous susceptible insects from the refuge mate with the resistant from the Bt crop field, their progeny will be heterozygous, meaning that they will have one susceptible allele, and one resistant allele. If this occurs effectively in the fields, the pest population will remain sensitive to the Cry toxin expressed in the Bt crop (Andow and Alstad, 1998).

Nevertheless, if two heterozygous insects mate, $\frac{1}{4}$ of their progeny will be resistant. For this reason it was suggested that in addition to the refuge strategy, the stacking of two or more *cry* genes that have different modes of action has been widely used to delay the evolution of resistance to Bt crops. For example, the stacked MON-88913-8 X MON-15985-7 event expresses the Cry1Ac and Cry2Ab toxins, which have been shown to have a different mode of actions, as they recognize distinct protein receptors in the guts of the same sensitive larvae (Caccia et al., 2010). The consequence that Cry1Ac and Cry2Ab recognize different proteins in the target pests, greatly reduces the probability of having a pest with double mutation (Caccia et al., 2010).

The eradication program of *P. gossypiella* implemented in the United States and Mexico since 2002 established the use of Cry toxins in conjunction with other control strategies. The adoption of Bt cotton with dual toxins by the local farmers resulted in the dramatic decline of this insect and its practically eradication in the Northern region of Mexico (SAGARPA, 2012, 2016; Martínez-Carrillo, 2015).

However, the secondary lepidopteran pest, *S. exigua* shows low susceptibility to Cry1A and Cry2A toxins, and recently it is causing significant damages to the Bt cotton crop in Mexico. To overcome this issue a new stacked event containing the *vip3Aa* gene plus *cry1A*, and *cry2Ab* (Kurtz et al., 2007; Carrière et al., 2015) might be deployed. Vip3A is a highly effective Bt protein that exhibits high toxicity against *S. exigua*, and it has a different mechanism of action than Cry proteins, thus these new pyramided events expressing also Vip3A could effectively control *S. exigua* (Lee et al., 2003; Chakroun et al., 2016). Therefore, this new stacked-Bt cotton variety has a wider spectrum of control than the previous ones, and it will be very helpful in insecticide resistance management. However, due to the high usage of Bt cotton in the American continent, the eventual evolution of resistance, even to the newly stacked events, cannot be ruled out. Therefore, it is necessary to continue searching for novel insecticidal proteins with different modes of action and high efficacy against different cotton pests.

#### Evolution of resistance in weeds

Regarding resistance to herbicides, the first cotton events used in Mexico and elsewhere contained glyphosate resistance genes, which caused an intense use of this herbicide in fields of GM cotton in very large areas of the planet, with the consequence of the evolution of resistance to glyphosate by a diversity of weeds (Powles, 2008). Currently, there are 40 weed species already resistant to glyphosate (Heap, 2018). For this reason, it is recommended the use GM cotton resistant to alternative herbicides with different mechanism of action and other integrated weed management practices that would allow an effective control of weeds, avoiding the evolution of herbicide resistance.

It is interesting that in Mexico there are no reports of weed resistance to the herbicides used in GM cotton (SENASICA, 2016; Heap, 2018). This may be due to the fact that Mexican cotton farmers commonly use conventional tillage and in-row cultivation. Adoption of no-tillage systems in herbicide-resistant GM crops seems to be part of the problem of evolution of herbicide-resistant weeds in countries such as USA, Brazil, and Argentina (Powles, 2008). To cope with this problem there has been a worldwide request to release events that have more than one gene of resistance to different herbicides such as ammonium glufosinate and glyphosate or glyphosate and dicamba.

Thus, in Mexico, the deep tillage along with manual removal of early weeds, in-row cultivation, and crop rotation have apparently delayed the appearance of glyphosate-resistant weeds despite the fact that GM cotton technology has been adopted for more than 15 years (CIBIOGEM, 2018). In contrast, in the United States the first case of Palmer amaranth *A. palmeri* resistant to glyphosate was reported in 2005 (Culpepper et al., 2006), only 8 years after this technology was adopted (Norswhorty et al., 2016).

## Conclusions, recommendations, and perspectives

*G. hirsutum* is a native species in Mexico, from which several highly efficient GM cultivars have been developed for the production of cotton worldwide, and some of them are now used in the north region of Mexico.

The tetraploid cotton *G. hirsutum* has a relatively large genome and diverged from its diploid ancestors several million years ago (Shan et al., 2016). Due to the distribution and chromosomal composition of this species, it is expected that there is low risk of introgression or mixing with other diploid wild species of Mexico by pollen flow, but seeds represent an important risk. However, it is still possible that the mixing of GM cotton with wild populations of the same species or another tetraploid specie occurs. It is also possible that the effect of this introgression may be diluted in the wild by processes like meiotic drive or by the lack of selective pressure to maintain the GM genes in complex communities and if the GM genes represent a cost to carry and to express them. Nevertheless, direct experiments will be required to follow the introgressed plants for several generations in the field. Also, given the possibility of introgression is a potential risk, careful monitoring programs for transgenes should be maintained, in particular focusing on the fate and dispersal of the seeds due to spills that occur during transportation from the fields to the gins.

We need detailed socioeconomic studies, as well as epidemiological studies on the health of Mexican cotton farmers, as nowadays there is not enough data to conclude on those aspects.

So far no cases of weed resistance to glyphosate associated with cotton have been reported in Mexico (Heap, 2018). However, it is strongly recommended to encourage the use of appropriate management practices and alternative herbicides with different mechanism of action to delay the evolution of resistance to glyphosate (Devine et al., 1992). In cases of resistance, the use of GM-glyphosate resistant seeds should be avoided since there is a greater danger of increasing the populations of glyphosate-resistant weeds species. This has already occurred in the United States, where weeds such as Palmer amaranth, Johnson grass and barnyard grass are now resistant.

It is known that the use of herbicides with two or more modes of action significantly delays the evolution of herbicide resistance (Neve et al., 2011). Besides, it is necessary to continue integrating the use of herbicides with other management practices, such as deep tillage, in row cultivation, and crop rotation to diversify weed management and decrease selection pressure for herbicide resistance.

The impact of Bt cotton on the use of chemical insecticides has been significant. Since its introduction 20 years ago, there has been a decrease in the use of chemical insecticides, but the data varies between regions due to differences in the ecological and management conditions, different composition of pests and other non-target pests. The evolution of resistance in target-pests cannot be ruled out, even despite the proper use of refuges.

The reduction in the number of applications ranges from one application in Sonora and Mexicali, to almost five applications of chemical insecticide per crop cycle in La Laguna. Also, it is important that the chemical insecticides that are currently used to control the pest complex have, in average, a lower environmental impact than the ones used a couple of decades ago.

Despite the relative good news, it is necessary that farmers and cotton technicians continue to get involved in the detection of a possible loss of efficacy of Bt cotton against the target pests. It is very important also to maintain the active participation of farmers and technicians for the prevention of the evolution of resistance, particularly in the adequate implementation of refuge areas.

In the future, the integration of various pest management tactics will be important, such as cultural control through the destruction of crop residues and biological control through the use of natural enemies (entomopathogens, predators, and parasitoids). The monitoring of insect resistance to Cry toxins expressed by the approved cultivars and those that are envisaged for their introduction in the Mexican market should continue. Federal support for cotton producers is considered crucial to continue with the Binational (Mexico-USA) Program for the eradication of *P. gossypiella* and *A. grandis*, in several regions to declare more free zones in a short term.

The change in the composition of primary insect pests and the increasing possibility of the development of glyphosate-resistant weeds, suggest the urgent need of developing new biotechnological tools to meet national needs. Policies directed toward federal funding for scientific research in Mexico, as well as a national program of seed production should be also strongly encouraged. Mexico has now the human and scientific capabilities and consistent funding of long-term goals directed to a more sustainable agriculture is needed. This is particularly important due to the lack of possibilities for producers, since there is no national policy for seed production, which puts at risk not only cotton, but also the national food security. Today Mexico depends totally on seeds from the large international companies for its cotton production.

Mexico has been careful in observing the principles of the Cartagena Protocol and the national regulation is highly demanding and expensive to meet. However, in many cases these regulations can only be met by the large companies; as a result, researchers and national institutions with low budgets find impossible to comply with all the requirements established in the biosafety law.

Finally we strongly recommended the agricultural and scientific authorities of Mexico to support a healthy long-term program of national research in order to meet the new needs of agriculture, conventional or GM, for the next 20 years.

## Ethics statement

Either ethics approval or written consent are not necessary in our study because it is not a clinical study, but instead shows a collection of technical opinions of a group of experts and did not involve disclosure of sensitive personal data. According to the Declaration of Helsinki, medical research is subject to ethical standards that promote and ensure respect for all human subjects and protect their health and rights and it is addressed primarily to physicians. In this work the information obtained from technicians and farmers is related to the crop cultivation practices, and it did not involve any medical study.

## Author contributions

MR-M, VS, and AB coordinated the research. LE and MS planned the obtainment of the data. EN, SC, ES collected data and apply surveys. DM-S, ER-R, UN-C, JM-C, CB, MR-M, EN, and SC designed the technician's survey. SC and ES performed the geographical modeling. All authors contributed with the data analyses, discussion of the results, and writing of the paper.

### Conflict of interest statement

The authors declare that the research was conducted in the absence of any commercial or financial relationships that could be construed as a potential conflict of interest. The reviewer JI and handling Editor declared their shared affiliation.
